# Desktop Fabrication of Lab-On-Chip Devices on Flexible Substrates: A Brief Review

**DOI:** 10.3390/mi11020126

**Published:** 2020-01-23

**Authors:** Ahmad Zaman Qamar, Mohtashim Hassan Shamsi

**Affiliations:** Department of Chemistry & Biochemistry, Southern Illinois University, 1245 Lincoln, Carbondale, IL 62901, USA; azqamar@siu.edu

**Keywords:** lab-on-chip, flexible devices, desktop fabrication, microfluidics, biosensors

## Abstract

Flexible microfluidic devices are currently in demand because they can be mass-produced in resource-limited settings using simple and inexpensive fabrication tools. Finding new ways to fabricate microfluidic platforms on flexible substrates has been a hot area. Integration of customized detection tools for different lab-on-chip applications has made this area challenging. Significant advancements have occurred in the area over the last decade; therefore, there is a need to review such interesting fabrication tools employed on flexible substrates, such as paper and plastics. In this short review, we review individual fabrication tools and their combinations that have been used to develop such platforms in the past five years. These tools are not only simple and low-cost but also require minimal skills for their operation. Moreover, key examples of plastic-based flexible substrates are also presented, because a diverse range of plastic materials have prevailed recently for a variety of lab-on-chip applications. This review should attract audience of various levels, i.e., from hobbyists to scientists, and from high school students to postdoctoral researchers, to produce their own flexible devices in their own settings.

## 1. Introduction

**Figure 1:**
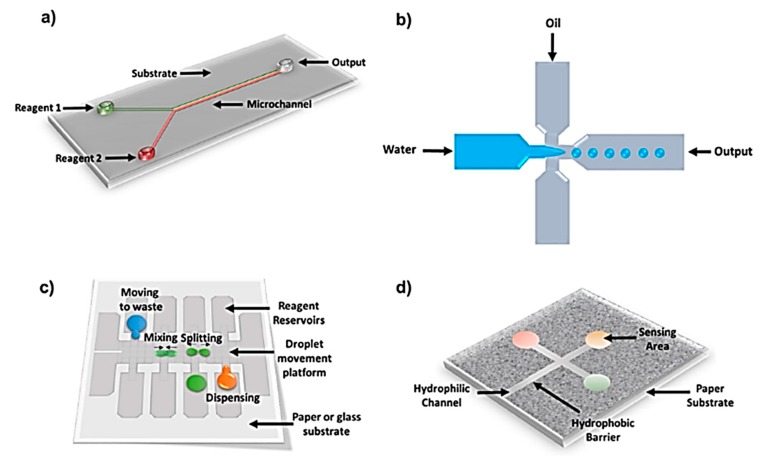
Schematics showing different types of microfluidic platforms: (**a**) Polydimethylsiloxane (PDMS) based microchannels showing a continuous flow channel microfluidic platform. (**b**) Droplet microfluidic system to create immiscible liquid droplets working as micro-reactors. (**c**) Digital microfluidic system where liquid droplets move under the influence of electrical potential. (**d**) Paper-based microfluidic system which works based on capillary action that moves liquid through hydrophilic channels (Adapted with permission from IOP Science) [1].

Miniaturized lab-on-chip (LOC) devices have the capability to perform laboratory procedures at a small scale. Performing multiple lab processes on a single chip with a minimum amount of reagents and high efficiency are the most promising features of these platforms. A variety of microfluidic systems have been developed thus far, as summarized in Figure 1, including channel-based, droplet-based, paper-based, and digital microfluidic systems. These microfluidic platforms, along with their applications and detection systems, have been comprehensively reviewed recently [1,2]. Despite compelling features and the future of LOC systems, the construction processes of many devices involve fabrication procedures that are complicated, expensive, and cumbersome [3]. The early development of miniaturized analytical platforms called micro total analysis systems (μTAS) involved molding, microcontact printing, micromachining etc. [4]. These multi-step and time-consuming methods were used to develop structures on a micrometer scale. With the passage of time, new methodologies were proposed wherein hard substrates were replaced with flexible substrates. Substrates made of paper and plastics offer unique characteristics, such as flexibility, durability, portability, low-cost, and simple fabrication due to compatibility with variety of printing tools. The market value of printed sensors on flexible substrates was around US $6.7 billion in 2015, and is expected to reach $10.5 billion in 2022, and potentially become one of the top five emerging fields in the following decade [5].

A recent review has comprehensively analyzed fabrication techniques of microfluidic paper-based analytical devices along with theoretical background, methods of fluid flow manipulation, and detection techniques [6,7]. In this short review, we present few representative examples of flexible devices fabricated by the most common desktop tools used independently or in combination on cellulose-based paper substrate [8,9], and also various plastic substrates that were reported in last 5 years [10,11,12,13,14,15].

### 1.1. Cellulose-Based Paper Substrates 

**Figure 2:**
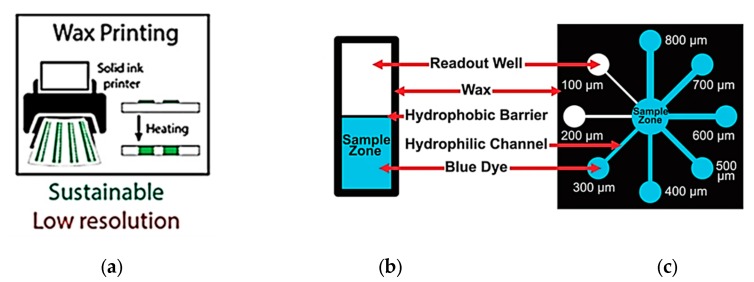
(**a**) Wax patterning on a paper substrate, followed by heating step, to create hydrophobic borders across the substrate. (**b**) A typical example of a µPAD device for the minimum hydrophobic barrier test. Black boundaries show printed wax which prevents liquid to flow out of channel. (**c**) A paper-based device with multichannel system of various widths (adapted with the permission from Creative Commons Attribution-Non Commercial-No Derivatives License) [17].

Paper is a promising flexible substrate for microfluidic applications because of its ubiquity, even in resource limited areas. In paper-based micromachines, liquid flows through paper-based channels under capillary action. Reagents are placed in a dispensing zone by a pipette and allowed to travel to a target zone of reaction where colorimetric response is prompted. Hydrophobic boundaries are created on paper-based substrates using wax material through screen or inkjet printing, followed by a heating step that allows wax to penetrate through the paper substrate and form hydrophilic channels. The flow of liquid relies on paper hydrophilicity, pore size, and direction of fibers. For instance, well-aligned fibers in the substrate accelerate fluid flow in the channels. The limitation of paper substrates is not every kind of paper can be used because of additives and unknown composition of other materials added during the manufacturing process. Therefore, mostly nitrocellulose and Whatman filter paper (1 and 4) have been widely employed, due to their known and reliable physical and chemical properties [16,17]. A standardized micro paper analytical device (µPAD) is shown in Figure 2 [17]. 

### 1.2. Plastic Substrates

**Figure 3:**
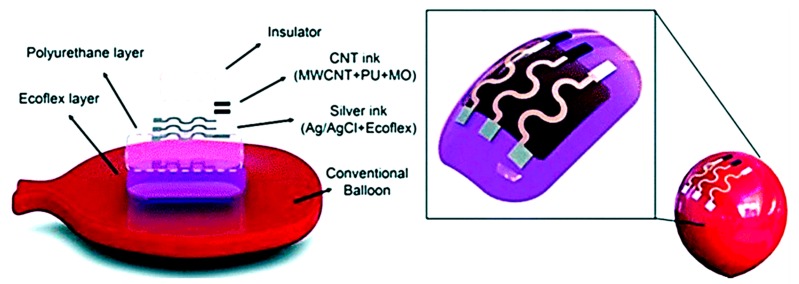
An example of wearable sensor demonstrated on a balloon which shows adaptability to curvilinear surfaces. The multiple layers consist of an Ecoflex layer (inner), polyurethane (intermediate), a printable sensor (Ag/AgCl with Ecoflex composite) and a flexible insulator layer (upper) (Adapted with the permission from Wiley Materials) [18].

Plastic-based flexible substrates are tough, heat resistant, and transparent in nature. Plastic substrates are widely used to pattern conductive materials for electrical and electrochemical sensing applications, such as wearable sensors [18]. Recently, Qamar et al. showed that when printing wax on polyethylene terephthalate (PET), heating is not required to create hydrophobic boundaries, in contrast to paper-based substrates, and the boundaries remain stable at a temperature as high as 160 °C because the PET substrates are nonporous and nonfibrous [13]. Moreover, the surface properties of the plastic substrates can be easily modified to make them hydrophilic to hydrophobic and vice versa [13]. The high thermal resistivity of several polymer substrates makes them suitable for applications where high temperatures are required during fabrication procedures. Table 1 lists several temperature resistant polymer-based substrates that were employed as device substrates for bioanalytical applications, including polyethylene terephthalate (PET) [10,11,12,13,14], polyethylene naphthalate (PEN) [19], polyimide (PI) [20,21], polyetheretherketone [22], polyether sulfone (PES) [23], polycarbonate (PC) [20,24,25], and polyester [15]. Figure 3 illustrates an example of wearable sensor demonstrated on a balloon which shows adaptability to curvilinear surfaces. The device consists of multiple layers comprising a Ecoflex silicon-based rubber layer (inner), a polyurethane layer (intermediate), a sensing layer of Ag/AgCl with the Ecoflex composite on polyurethane, and a flexible insulator layer (upper) [18].

## 2. Fabrication of Flexible Microfluidic Devices 

**Figure 4:**
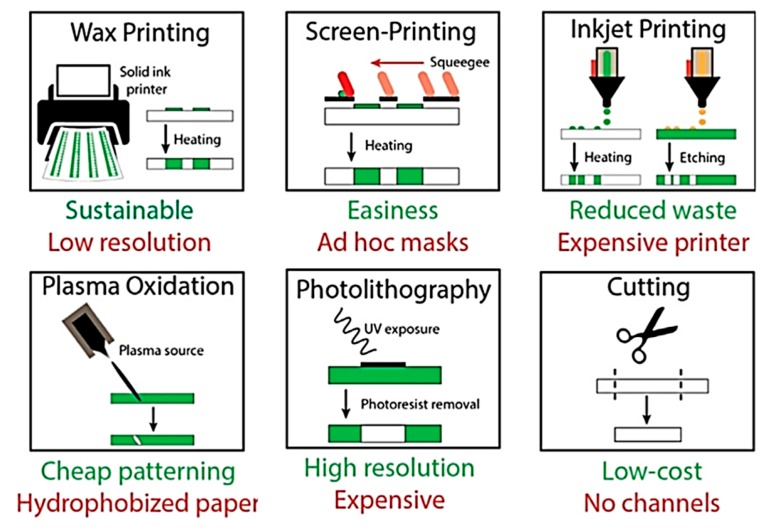
Types of fabrication methods to construct lab-on-chip devices (Adapted with the permission from Elsevier) [28].

In this section, we review number of low-cost, small footprint fabrication tools that are commonly used along with expensive photolithographic procedures to construct devices. The fabrication tools along with their pros and cons illustrated in Figure 4 can be used alone or in combination to fabricate a whole device [28].

### 2.1. Wax Dipping

**Figure 5:**
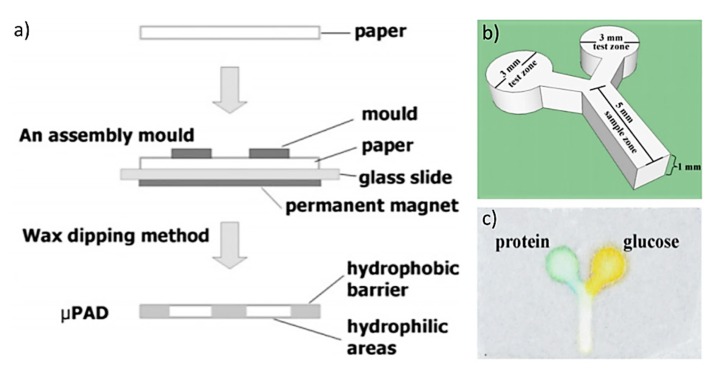
Wax dipping method and detection of analytes: (**a**) Procedure for patterning paper by wax dipping (**left**). (**b**) Shape and size of an iron mask. (**c**) Detection of protein and glucose on a paper microfluidic device by the wax dipping method (Adapted with the permission from Elsevier) [29].

Wax dipping is a process of creating hydrophobic wax boundaries by dipping a cellulose substrate into wax liquid. In this strategy, a mask is required to transfer a pattern. In a recent example, white beeswax pellets were heated first in a beaker at 120–130 °C to get molten wax. A reusable iron mask was cut and placed on a cellulose-based substrate. The mask (iron mold) was placed on top of a filter paper backed by a glass slide; then, the filter paper and glass slide were sandwiched between a magnet and the iron mold. The whole setup was dipped in the melted wax for a short moment at 120–130 °C. After cooling at room temperature, the filter paper was peeled off the glass slide with the removal of iron mask. The area covered by mask was turned into hydrophilic channels while the exposed area became hydrophobic, as shown in Figure 5. The highest resolution achieved by this method was 639 ± 7 µm [29]. Laiwattanapaisal’s group used this strategy to fabricate devices for several interesting applications that have potential for clinical applications. In one example, their group used the paper-based devices for the separation of blood plasma from whole blood and quantifying plasma proteins in a single step in 2 min with blood sample of 15–22 μL [30]. Recently, the same group used a combination of wax printing and wax-dipping to fabricate μPAD devices for simultaneous determination of Rh typing and forward and reverse ABO blood groups with a 10 min assay time. The assays were based on the ratio between the distance covered by red blood cell and plasma separation for agglutination, and indicate the presence of the corresponding antigen or antibody [31].

### 2.2. Solid Wax Printing

Hydrophilic patterns can also be printed on flexible substrates using a desktop wax printer that uses colored solid wax cubes as ink. The wax printer heats the wax cube to melt and jet out the molten wax to print patterns on a substrate. As mentioned earlier, these patterns are later heated on a hotplate or in an oven that allows the solid wax to penetrate through porous cellulose substrates, leading to the creation of a hydrophobic barrier. The resulting hydrophilic channels between the hydrophobic boundaries are robust but low resolution; i.e., 500 μm for smooth flow [32]. 

**Figure 6:**
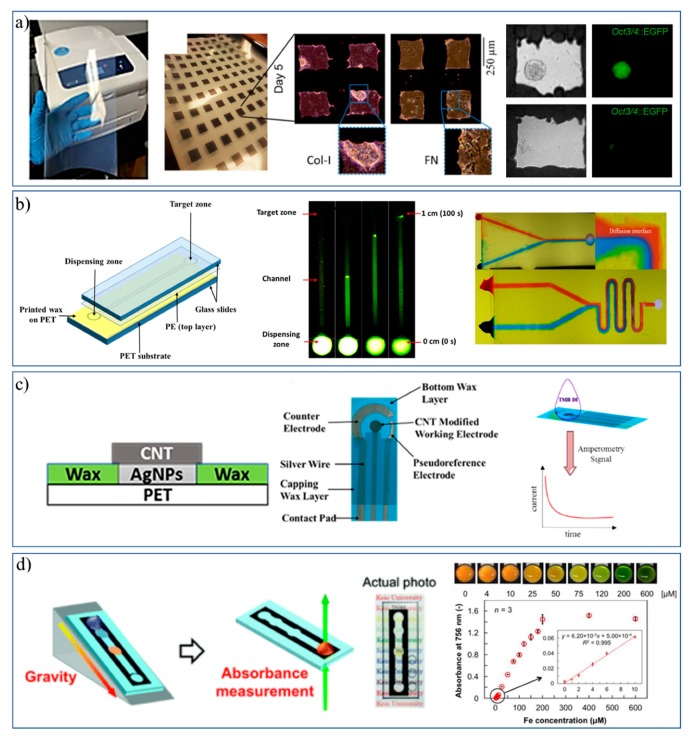
Wax on plastic platforms: (**a**) Wax printed microwells and stem cells applications. (**b**) Flow on fluorescently labeled DNA (**green**) and mixing of food colors (**red** and **blue**) in a wax-on-plastic microfluidic system. (**c**) Fabrication of a multilayer electrochemical sensor using a combination of wax and inkjet printing. (**d**) Device showing the gravitation flow of liquid through a wax printed channel on a transparent film for detection of Fe^2+^. (Adapted with permission from RSC and Springer Nature) [13,34,35,36,37].

Since 2009, solid wax printers have been widely used to print designed patterns onto cellulose-based substrates [17,33]. Recently, so called ‘wax-on-plastic’ platforms were developed which involved wax patterns on PET substrates. Various types of such platforms have been developed and employed for various applications by Shamsi’s group, as summarized in Figure 6. First, Qamar et al. explored the printability, fidelity, and application of wax micropatterns on a non-cellulosic, non-fibrous, and non-porous polyethylene terephthalate (PET) based substrate, where the printing resolution was found to be 60 µm for line patterns and 120 µm for channels with the advantage of thermal stability over melting temperature of wax; i.e., >120 °C [13]. Later, high density microwells were patterned on PET by this strategy and successfully used to study the fate of mouse embryonic stem cells (mESCs), i.e., renewal or differentiation, under microenvironment cues. The stem cells showed the distinct differentiation in fibronectin and self-renewal in collagen-I microenvironments with no sign of toxicity, as shown in Figure 6a [13]. 

Patterning of wax microchannels on flexible PET substrate directed the evolution of “wax-on-plastic microfluidics” that was used to detect glucose in urine by a flow-based assay [34]. The liquid flow in these channels is driven by capillary force due to the difference in surface energies between the substrate (hydrophilic) and the boundaries (hydrophobic), which allows one to perform a bioassay within 40 s at sub-microliter level. Recently, Qamar et al. discriminated neurodegenerative trinucleotide repeat sequences (TNR) of DNA using the same platform by monitoring the flow dynamic properties of such sequences that reflect their molecular conformation and structural properties [35]. The capillary flow of these sequences was found to be function of type, size, concentration, molecular conformation, and metal ion concentration dependent. The fluid flow in wax-on-plastic microchannels is laminar; that allows us to study diffusion between two liquids at the interface in V-shaped channel, Figure 6b (right). Serpentine channels can facilitate mixing of liquids without any external driving force. 

Chens et al. combined the wax printing with hand painting of conducting materials, i.e., AgNPs and CNTs, on PET substrate to develop the multilayered electrochemical sensor shown in Figure 6c. This fabrication was found to be convenient and simple for making the electrochemical sensor; it involved wax patterning on plastic, hand painting of AgNPs to lay down a conducting layer, and simple drop casting of CNTs to enhance electrochemical properties of working electrodes. The device layers were well-adhered to each other and the wax layer on plastic can easily sustain a sintering temperature of 120 °C. This reliable fabrication process was tested by amperometric current response and the CNTs/AgNPs sensors detected carcinoembryonic antigen with the limit of detection ca. 0.46 ng/mL, which is comparable to the performance of commercial screen-printed electrodes and recently reported paper-based electrochemical sensors [36].

Fugisaki et al. reported open channel wax printed microfluidic chip to develop transmittance-based assay to detect iron [37]. As shown in Figure 6d, the microfluidic chip was mounted on a 3D printed ramp to facilitate gravity assisted flow of liquid. In this example, the sensing zone was scanned using a colored scanner and the color intensities were compared using ImageJ software. Transparent plastic helped to visualize the microfluidic flow in channels and to develop absorbance/transmittance-based assays. Considering the robustness, low cost, convenience, high throughput fabrication of wax-on-plastic platforms, they have a potential to be adopted for developing variety of lab-on-chip micromachines.

### 2.3. Screening Printing

Screen printing is a traditional printing technique involving a customized mesh and used to transfer patterns onto a substrate. In this procedure, material (ink) is transferred selectively through a mesh and making some areas impermeable to the transferring material. A squeegee or a flat object can be moved back and forth over the screen to spread out liquid or slurry material and push the material onto a substrate [38]. Although a massive number of devices can be produced using screen printing, it requires a customized screen via photolithography to transfer pattern onto a substrate. The screens are reusable; however, making customized screens for each type of pattern is a time consuming and cost additive step of fabrication. Moreover, the feature sizes and spatial resolutions of screen-printed devices are large; i.e., in millimeter scale range [39].

### 2.4. Inkjet Printing 

**Figure 7:**
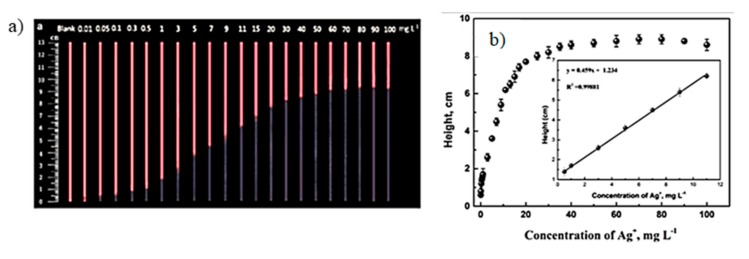
(**a**) Quenching of the fluorescence band in the presence of different concentrations of Ag^+^ in mg/L under UV light at 365 nm. (**b**) Corresponding calibration curve obtained in the presence of various concentrations of Ag^+^. (Adapted with permission from American Chemical Society) [45].

Inkjet printing is known as digital writing, which is used to deposit functional materials onto a substrate. It uses a non-impact technique for patterning of functional materials on pre-determined spots of substrates. Inkjet printing is a cost effective and widely adopted tool for the fabrication of microfluidic devices by depositing hydrophobic materials; e.g., alkyl ketene dimer, silicone, polystyrene, polyacrylate, and conductive inks [40,41,42,43]. Hydrophobicity can be patterned selectively onto a paper substrate with no heating required to create hydrophobic barriers across the paper substrate. The inkjet printable conductive materials include metallic inks, e.g., AgNPs, graphene, carbon nanotubes, and quantum dots, which are used to fabricate biosensors [44]. An interesting example involved detection of silver nanoparticles (AgNPs) that are released into the environment from consumer products and are potentially hazardous to health. To detect the AgNPs in consumer products, inkjet printing was used to pattern CdTe quantum dots on paper-based devices. When paper strips were dipped in the solutions containing silver ions, it would quench the fluorescence of CdTe quantum dots. The concentration of silver ions present in the sample was directly related to the height of the fluorescent band to get calibration curve, as shown in the Figure 7 [45]. There are numerous advantages of inkjet printing including mask-less technique, time efficient, has good reproducibility, and has high spatial resolution. The printing resolution depends on ink rheological properties, substrate porosity and surface energy, and size of printer nozzle. Nevertheless, one can print features of 10–20 μm width with spatial resolution of 10–15 μm using a materials grade printer, such as Dimatix [46,47]. However, major challenges of inkjet printing are compatibility of the surface energies of ink and substrate, rheological properties of the ink suitable for the printer’s head and nozzles, changes in the properties of printing material, and cleaning of the nozzles as a result of clogging.

### 2.5. Photolithography

Photolithography is known for fabricating devices with high resolution features ≈5 μm. It is a sophisticated and cumbersome procedure that requires highly skilled personnel and several steps. The typical steps involve vigorous substrate cleaning, application of photoresist, baking, UV-light exposure, etching, and developing of features by removal of photoresists. In brief, patterns are made by shining a UV-light on a substrate covered with a photoresist and a patterned mask. When light passes through a mask, it selectively creates patterns onto a substrate by curing an exposed photoresist, and the uncured area is later developed for subsequent steps. Whiteside’s group showed the first examples of paper-based devices produced by photolithographic procedures, such as paper-based devices for real-time diagnosis by cell-phone [48], paper-based microzone plates [49], and a rapid method for laboratory prototyping of microfluidic devices in paper [50]. Moreover, plastic substrates are widely used to map high resolution (<100 nm) conductive patterns for organic field effect transistors [51], and optoelectronic devices [52]. Despite the advantage of high resolution, photolithography did not gain popularity for resource-limited settings, because it is expensive, and requires large-footprint equipment, a special environment, and highly pure reagents for the procedure [53].

### 2.6. Desktop Cutter

**Figure 8:**
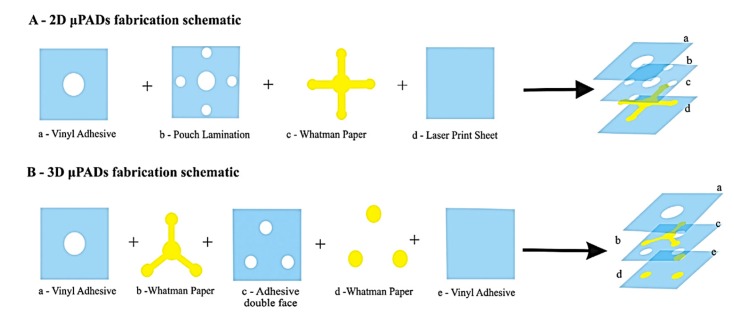
Fabrication of 2D and 3D µPADs through vinyl cutter (Adapted with the permission from Elsevier) [57].

Desktop cutter is an inexpensive and portable tool that can create microchannels down to 200 μm on flexible plastic substrates using a programmed sharp needle [54]. This tool can also be used to develop a 3D microfluidic system, and a recent example employed Parafilm^®^ film which was cut and laminated with a transparent, thin poly(ethylene terephthalate) film to build versatile two and three-dimensional microfluidic chips [55]. This tool is also known as a vinyl cutter because it is frequently used to cut adhesive-backed patterned vinyl sheets to make screens for patterning electrodes, such as by hand screen printing [56]. Figure 8 shows the fabrication of inexpensive sealed 2D and 3D µPADs for the analysis of glucose, total protein, and nitrite on single device [57]. In brief, a cellulose filter paper with central sample loading zone and branched detection zones were sealed between a polyester sheet and a laminating pouch followed by heating at 120 °C and compressed for 120 s with heat press machine for safe handling and transportation [57]. The disadvantage of vinyl cutter method is that the minimum feature sizes are usually several hundred microns.

### 2.7. Laser Cutter 

A laser cutter uses high intensity CO_2_ lasers to create hydrophilic patterns on variety of materials from ceramics and glass to plastic and papers. In paper-based fabrication it creates hydrophilic features on hydrophobic papers, e.g., parchment or wax paper, with a resolution of up to ≈62 µm, whereas wax barriers on cellulose paper have resolution of 100–300 µm [58]. Nie et al. reported a laser cutting method to fabricate microfluidic μPADs by patterning hollow microstructures in paper as having channel features of 500 µm [59]. Thompson et al. used a protocol called laser print, cut, and laminate (PCL) methodology. Microfluidic devices with complex multilayer architectures were fabricated using a laser printer, a CO_2_ laser cutter, an office laminator, and common overhead transparencies (as a printable substrate) [60].

### 2.8. Desktop Pen-Plotter

**Figure 9:**
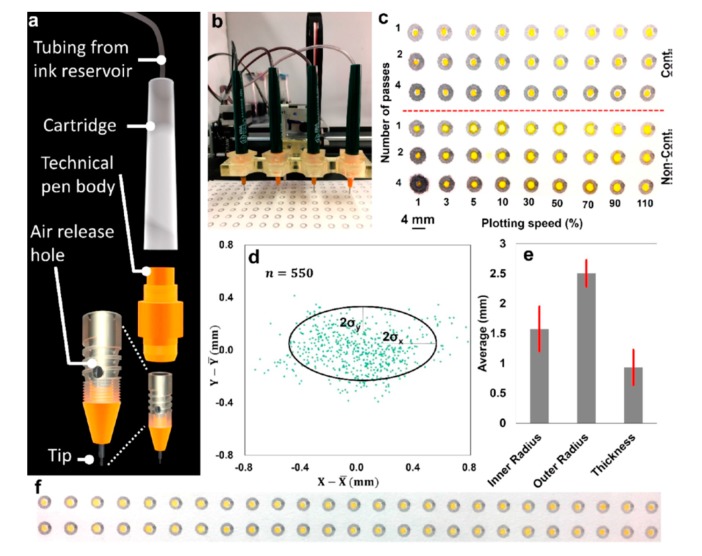
(**a**) Illustration of the continuous ink technical pen. (**b**) Technical pens fed continuously by ink. (**c**) The effects of different plotting speeds and numbers of passes on the water resistance of patterns in a continuous (**top**) and noncontinuous (**bottom**) plotting system. (**d**) Deflection of the plotted patterns in both the x and y directions. (**e**) comparison of the dimensions of the printed spots by the pen-plotter. (**f**) A representative image shows the performance of the continuous plotting system in high-throughput fabrication of paper-based microfluidics. (Adapted with the permission of American Chemical Society) [63].

The pen-plotter has been around for around four decades; it draws programmed vector graphics on paper using a pen [61]. Recently, Tasoglu group used the pen-plotter-based approach to study the performance of variety of commercial permanent markers to draw hydrophobic features on paper substrates. The microfluidic devices prepared from this approach were used for proof-of-concept colorimetric detection of glucose [62]. The same group used 3D printed multiple-pen holder to customize the pen-plotter for developing a continuous-ink, multiplexed pen-plotter for low-cost, high-throughput fabrication of paper-based microfluidics, as shown in Figure 9. The plotting performance of the continuous plotting system shows that the deflection in both x and y axes was 400 μm (Figure 9d), while the feature’s size was in millimeter range (Figure 9e). A potential application was colorimetric urine assays for nitrite, urobilinogen, protein, blood, and pH [63]. 

## 3. Combination of Fabrication Tools

The fabrication tools discussed above have also been combined to improve fabrication efficiency, lower the cost of fabrication, and implement unique features in the devices. In this section, we review recent examples of devices that were fabricated by the combination of various tools discussed above.

### 3.1. Wax and Screen Printing 

**Figure 10:**
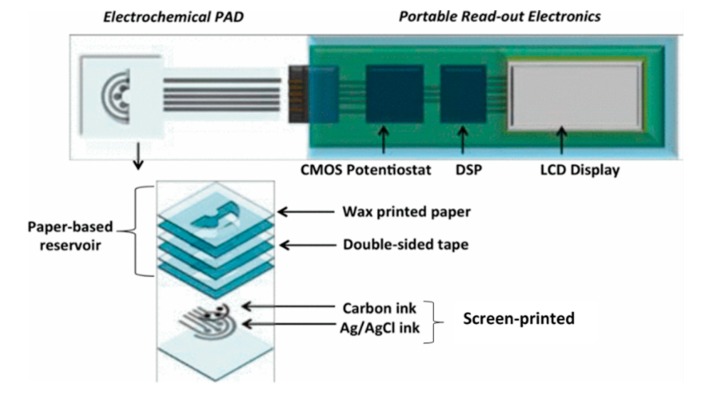
3D electrochemical origami paper-based analytical device (omPAD) integrated with a custom CMOS potentiostat (Adapted with the permission from IEEE) [67].

A typical combination of wax printing and screen printing involves the creation of hydrophobic barriers of wax and the patterning of electrochemical sensors in hydrophilic regions of a substrate by screen printing [64]. Recently, several interesting approaches emerged that include fabrication of bipolar electrochemiluminescence (BP-ECL) biosensors on paper [65], a three-dimensional cloth-based microfluidic ECL biosensor, and an integrated microfluidic paper-based analytical device with a three electrode system (working, counter, and reference) and screen printing [66]. Figure 10 illustrates a 3D origami paper-based analytical device (omPAD) with multiple electrochemical sensors, an integrated sample reservoir, and tight integration with a custom CMOS potentiostat. The device layer shows the combination of paper-based reservoir stacked by wax-printed paper and double-sided tape, while the screen-printed carbon electrodes (working and counter) and Ag/AgCl reference the electrode [67].

### 3.2. Wax Printing and Laser Cutter

**Figure 11:**
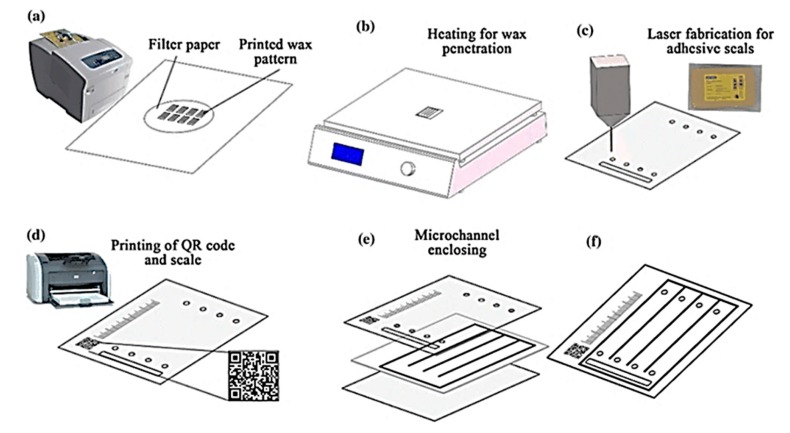
Schematic of the microchannel fabrication and microchannel enclosing using wax printing and laser cutter where (**a**) printing wax pattern on a fiter paper, (**b**) heating step to penetrate wax through paper, (**c**) laser fabrication for adhesive seals, (**d**) inkjet printing of QR code and scale (**e**) enclosing microchannels by combining device layers, and (**f**) final device construct. (Adapted with the permission from Springer Nature) [68].

A combination of wax printing and laser cutting was employed to fabricate fully enclosed paper-based microfluidic devices with bio-compatible polyester adhesive seals. The bonding strength between adhesive seal and filter paper was also tested in the proposed microchannel enclosing method [68]. Figure 11 illustrates the wax patterning on a paper substrate creating hydrophobic wax barriers in circular fashion (70 mm) using a desktop wax printer. After baking, inlet and outlet holes were created with a CO_2_ laser cutter. The top and bottom of the wax printed chip were covered with an adhesive seal to enclose the device. The paper-based device was used to analyze amino acids by distance-based assay where scale bar and QR code were also printed to measure the relative distance of color of analytes appeared on the chip [68]. 

### 3.3. Screen Printing and Laser Cutter 

**Figure 12:**
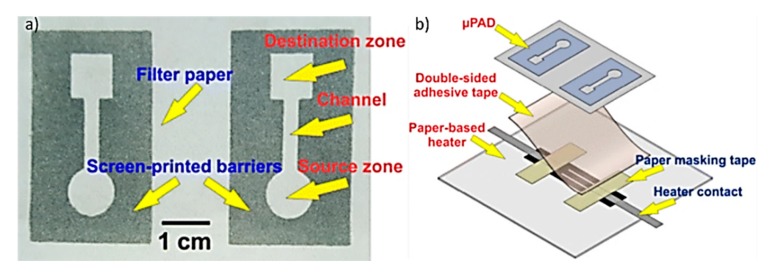
(**a**) Image of paper-based µPAD, which is fabricated by screen-printing of crayon eye liner on filter paper. (**b**) Schematic shows the process of sticking the µPAD to the paper-based heater using a piece of double-sided adhesive tape (Adapted with the permission of Springer Nature) [69].

A paper-based microfluidic temperature sensor was fabricated by combination of screen printing and a laser cutter, as shown in Figure 12. Screen-printed, identical patterns on paper were adhered to a heater by a piece of double-sided adhesive tape in such a way that only one of the patterns was placed on the heating area. The heater was adjusted to 37 °C by the proportional integral derivative (PID) controlling system. Two droplets of cholesterol were dropped on the square areas (destination zones) of the µPAD. The volume of the droplets was not large enough to pass through the channels. Subsequently, two droplets of cholesterol standard serum (10 µL) were dropped on the circular areas (source zones). The serum moved through the channel from the source to the destination and mixed with the cholesterol kit. A piece of transparent scotch tape was then stuck on the µPAD to protect the mixture from evaporation. The reaction between the serum and the kit gradually turned the color of destination zone to pale red [69,70].

### 3.4. Laser Cutter for Paper and Plastic Combination

**Figure 13:**
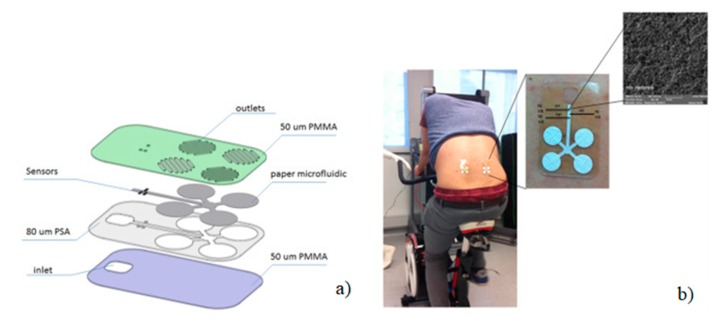
Schematic representation of the fabrication steps of the micro-fluidic chip on PMMA where (**a**) device layers, assembly and application on human sweat monitoring and (**b**) sensor structure and surface (Adapted with the permission of Elsevier) [71].

A recent report used a substrate combination to fabricate LOC devices where human sweat analysis was performed on an integrated sensing chip comprising a hybrid of poly(methyl methacrylate) and paper. A laser cutter was used for cutting the substrates to form microfluidic assembly consists of layers of micropatterned polymer and paper substrates. Sensors were placed inside the microfluidic channel which draws a constant flow of fresh sweat passing through these channels, as shown in Figure 13a [71]. In brief, the microfluidic patch consisted of layers of polymer, paper microfluidics, and flexible sensors. A CO_2_ laser was used to cut the structures on various polymer layers, which included a thick layer (80 µm) of pressure sensitive adhesive (PSA) that was laminated onto a layer of 50 µm thick poly(methyl methacrylate) (PMMA). The sensors were placed inside the microfluidic channel, which allowed a constant flow of fresh sweat to pass through the sensors. The final device was a hybrid microfluidic device produced by simply incorporating paper structures inside the microchannel of the platform to ensure flow of fresh sweat from the skin surface towards the electrochemical sensors through capillary action. The capillary flow was controlled by incorporating two different grades of paper. The system was used to simultaneously and selectively measure lactate, sodium, and pH, along with temperature sensing for internal calibration. The construction of the platform is designed such that continuous flow of sweat can pass through an array of flexible microneedle type of sensors (50 µm diameter) incorporated in a microfluidic channel. Potentiometric sodium ion sensors were developed using a polyvinyl chloride (PVC) functional membrane deposited on an electrochemically deposited internal layer of poly(3,4-ethylenedioxythiophene) (PEDOT) polymer (Figure 13b). 

## 4. Conclusions and Future Directions

The growing demand of low-cost lab-on-chip devices for healthcare applications in resource-limited settings has pushed the boundaries of the field of device fabrication. In this short review, we reviewed the fabrication of flexible devices on paper and plastic substrates using the desktop fabrication tools that are easily affordable, can be used individually and in combination, and are workable with less trained hands. The field of low-cost healthcare sensing and biosensing platforms is rapidly growing, and the desktop tools may be used to generate customize devices by end users or in clinics. The potential of plastic-based substrates will lead to reuse of old devices by recycling plastic-based materials. The progress in the field certainly needs a comprehensive review covering details on the performance, ruggedness, and limitations of the devices created by such low-cost tools. Here, we have exposed our readers to recent developments so that they can find opportunities from these examples for their desired applications. 

In terms of future directions, the area of flexible microfluidic micromachines will continue to grow in plastic-based substrates due to their versatile and superior properties over paper-based substrates. The recent examples of wax-on-plastic devices have provided an alternative to paper-based devices to overcome the limitations. Other advantages of plastic-based materials include tunable chemistry of plastics and the ability of surface functionalization which reinforces the application of plastic substrates for electrical, electrochemical, colorimetric, distance-based, and fluorescence-based assays. Another piece of progress in the area is the construction of hybrid systems that combine paper and plastic materials to perform respective functions. Interestingly, the low-cost and ease of operation of the desktop fabrication tools have now made them available for public science education at places such as libraries and science centers, which promises the conception of unique devices in future.

## Figures and Tables

**Table 1:** Flexible substrates incorporated in lab-on-chip devices [26,27].

Plastic Substrates	Thermal Stability (°C)	Analyte	Limit of Detection
Polyimide (PI)	250–300	Piperacillin	2.07 ng/mL
		Tetracycline	6.33 ng/mL
Polyethylene terephthalate (PET)	250–260	Uric acid	3 × 10^−9^ mol/L
		Lactate	1.0 µmol/L
Polyamide (PA)	190–350	Interleukin-6	0.2 pg/mL
		Cortisol	10 ng/mL
Polycarbonate (PC)	260–270	Lactate	NR
Polyethylene naphthalate (PEN)	185–200	C-MYC gene, Lactate	NR
